# Biomimetic Organic-Inorganic Hybrid Membranes for Removal of Fluoride Ions

**DOI:** 10.3390/ma15103457

**Published:** 2022-05-11

**Authors:** Yun Chen, Hao Kong, Lei Guo, Gang Wei

**Affiliations:** 1 College of Chemistry and Chemical Engineering, Qingdao University, Qingdao 266071, China; chenyunwu823@outlook.com (Y.C.); konghao5@outlook.com (H.K.); 2 Institute of Biomedical Engineering, College of Life Science, Qingdao University, Qingdao 266071, China

**Keywords:** biomimetic synthesis, peptide nanofibers, carbon nanofibers, hybrid membrane, water purification, sustainability

## Abstract

Carbon nanofibers (CaNFs) exhibit promising applications in the fields of environmental science and nanotechnology, and self-assembled peptide nanofibers (PNFs) are useful for the biomimetic synthesis of organic-inorganic hybrid nanomaterials and the fabrication of functional hybrid membranes for the removal of various pollutants from water. In this work, we report the biomimetic synthesis of hybrid nanomaterials by the interweaving of CaNFs and PNFs. Using the biomimetic mineralization properties of PNFs, ZrO_2_ nanoparticles were synthesized along the nanofiber surface, and then functional nanohybrid porous membranes were prepared by the vacuum filtration technology. For the fabrication of membranes, the amount of PNFs and ZrO_2_ precursors in the hybrid membrane were optimized. The designed organic-inorganic hybrid membranes exhibited high removal performance for fluorine ion (F^−^) from water, and the removal efficiency of the fabricated membranes towards F^−^ ion-containing aqueous solution with a concentration of 50–100 mg/L reached more than 80%. In addition, the nanofiltration membranes revealed good adsorption capacity for F^−^ ions. It is expected that the strategies shown in this study will be beneficial for the design, biomimetic synthesis, and fabrication of nanoporous membranes for economic, rapid, and efficient water purification.

## 1. Introduction

Fluorine is the most active element that exists widely in the form of fluoride (F^−^) in soil, rock, water, animals, and plants [1]. F^−^ in drinking water can effectively reduce the incidence of dental caries, but excessive fluoride intake can lead to many diseases, such as enamel fluorosis, skeletal fluorosis, Alzheimer’s disease, and thyroid disease [2]. F^−^ mainly comes from geochemical processes and industrial production processes [3]. The reversible dissolution/complexation of fluorine in ores and aquifers can lead to regional fluorine pollution, and with the rapid development of modern technology and industry, a large number of fluorine-containing pollutants will be produced in the process of industrial production. For instance, the concentration of fluoride ions recorded for rift valley lakes such as lake Shala and lake Abijata were found to be 246 mg/L and 204 mg/L [4]. In addition, the concentration of F^−^ in groundwater in Rome is 2.8 mg/L [5]. The World Health Organization (WHO) stipulated that the content of F^−^ in the drinking water must not exceed 1.5 mg/L, so it is very important to solve the F^−^ pollution in the water environment. At present, the commonly used technologies for the removal of F^−^ include the ion exchange [6], chemical deposition [7], photocatalytic degradation [8], reverse osmosis [9], and reverse electrodialysis technique [10]. Previous studies have indicated that these traditional techniques exhibited a few problems, including high energy consumption, relative low performance, and complicated operation process, in some way limiting real applications in high throughput treatment and purification of industrial wastewater.

Compared with the above-mentioned water purification methods, the membrane filtration has attracted more attention because of its simple operation, easy preparation, low energy consumption, and high removal efficiency [5,11]. The reasonably designed membrane materials are expected to become one of the optimal solutions for the removal of F^−^ from wastewater [5,12]. The nanomaterials commonly used to prepare filtration membranes are cellulose [11], graphene [13], carbon nanotubes (CNTs) [14], carbon nanofibers (CaNFs) [15], and many others. Among them, CaNFs are the kinds of functional carbon nanomaterials with porous, hollow, helical, and stacked cup structure, which have the advantages of low density, large specific surface area, small number of defects, high mechanical strength, and high electrical conductivity [16,17]. Unlike easily stacked graphene and CNTs, CaNFs can be easily dispersed in solution with increased mechanical strength, and their production is much simpler and cheaper [18]. In addition, there are many methods for the chemical modification of CaNFs [19,20]. For example, Rasheed et al. studied the oxidation of CaNFs by different oxidants (HNO_3_, KMnO_4_, RuO_4_, and concentrated H_2_SO_4_/HNO_3_ mixed oxidants) [21], and compared the yield and degree of carboxylation of these oxidants. Based on these advantages, CaNFs have shown broad application prospects in the field of nanotechnology, energy storage, and environmental science [22,23,24,25]. For instance, Beck et al. used lignin and polyacrylonitrile (PAN) as raw materials to prepare CaNF-based membranes by electrospinning technology. The fabricated CaNf-based materials exhibited high adsorption ability for methylene blue (MB) in wastewater [26]. In another study, Kumar et al. prepared CaNF/TiO_2_-polyacrylonitrile hybrid membranes by electrospinning technology to effectively remove metal ions and cationic dyes from wastewater [27].

Recently, metal oxides as adsorption substrates or dopants have become a research hotspot in the treatment of environmental pollutants [28]. A previous study has proved that Zr (IV) showed high binding ability and affinity towards F^−^ ions through the formation of a coordinated complex [29]. Compared with other F^−^-removing metal oxide materials (such as MnO_2_, TiO_2_, and CeO_2_, etc.), ZrO_2_ has the characteristics of low cost, great safety, environment-friendly, high chemical stability, and strong adsorption capacity for F^−^ ions [30]. Loading ZrO_2_ nanoparticles (NPs) onto the nanofibers to achieve efficient adsorption of F^−^ is a potential strategy for the removal of F^−^. The design and preparation of self-assembled peptide nanostructures make it possible to create functional nanomaterials for various applications [31,32]. In addition, peptides are excellent candidates for sustainable water purification materials due to their diverse functional groups and rich binding sites for various ions [33,34,35]. In addition, self-assembled peptide nanofibers (PNFs) as biomaterials have the unique characteristics of metal ion binding and biomimetic synthesis of metal oxide nanomaterials [36,37]. The exposed amino acids on their surfaces are closely bound to Zr (IV) due to electrostatic interaction and complexation. This method is simple, convenient, green, safe, and low energy consumption, but it requires the use of a high quantity of peptides.

Previously, many researchers have used other relatively cheap substances as substrates to facilitate the fabrication of macroscopic membranes with reduced cost. For example, Mezzenga et al. reported a new strategy that relies on carbon hybrid membranes made of amyloid fibers and ZrO_2_ NPs, which could effectively remove F^−^ ions from polluted rivers [5]. For the F^−^ ions with both low and high concentrations (about 200 mg/L), the fabricated membranes had a removal efficiency of more than 99.5%. This work hopes to develop a more economical hybrid membrane to purify F^−^-containing wastewater by optimizing the synthesis method. Therefore, we choose the low-cost CaNFs as the main support substrate to prepare the filter membrane, so as to further reduce the cost and promote the process of industrialization.

In this work, we propose biomimetic mineralization on the nanohybrid composed of CaNFs and PNFs, and the fabrication of functional two-dimensional (2D) nanofiber membranes via filtration under vacuum. We first study the self-assembly of peptide molecules with a sequence of KIIIIKYWYAF and tailor the formation of morphology-uniform PNFs by adjusting the reaction conditions of peptide molecules. The nanofiber hybrid material (CaNF/PNF) is then successfully prepared by mixing chemically modified CaNFs with PNFs solution, and then ZrO_2_ NPs are formed by controlled biomimetic mineralization on the CaNF/PNF hybrids. In addition, the amino groups and negatively charged groups on CaNFs and PNFs provide more binding sites for the formation of nanoparticles, so that the ZrO_2_ nanoparticles can have a good adsorption performance towards F^−^. Finally, a CaNF/PNF-ZrO_2_ nanofiber hybrid membrane is prepared by vacuum filtration. The purpose of this paper was to design a nano-hybrid membrane with a simple preparation method and good adsorption performance for F^−^ in wastewater, so that it can exert great potential in the field of environmental science.

## 2. Materials and Methods

### 2.1. Materials and Reagents

The KIIIIKYWYAF peptide was provided by the SynPeptide Biotechnology Co., Ltd. (Nanjing, China). HNO_3_ (purity 10%), N-hydroxysuccinimide (NHS, purity 98%), N-ethyl-N′-(3-(dimethylamino) propyl) carbodiimide (EDC, purity 98%), morpholine ethane-sulfonic acid (MES, purity 99%), and ZrOCl_2_⋅8H_2_O (purity 98%) were provided by the Rhawn Reagent Corporation Company (Shanghai, China). NaF (purity 98%) was used for the preparation of F—containing water samples, which were bought from the Macklin Company (Shanghai, China). Other chemicals, such as NaOH (purity 96%), trifluoroethanol (TFE, purity 99.5%), trifluoroacetic acid (TFA, purity 99%), and HCl (purity 36–38%) were provided by the Sinopharm company. (Beijing, China).

### 2.2. The Functionalization of CNFs

Firstly, 0.1 g CaNF solid was oxidized with concentrated HNO_3_ at 70 °C for 6 h, during which carboxyl groups were formed on the surface of the graphite layer of CaNFs. Then the oxidized carbon nanofiber (oCaNF) solid was washed and filtered by the vacuum filtration technology, and the black solid was washed thoroughly with ultra-pure water to clean the excess reagents, and finally to make the pH value of the filtered solution to about 7. Finally, the obtained black solid of oCaNFs was collected for next modification.

**Figure 1:**
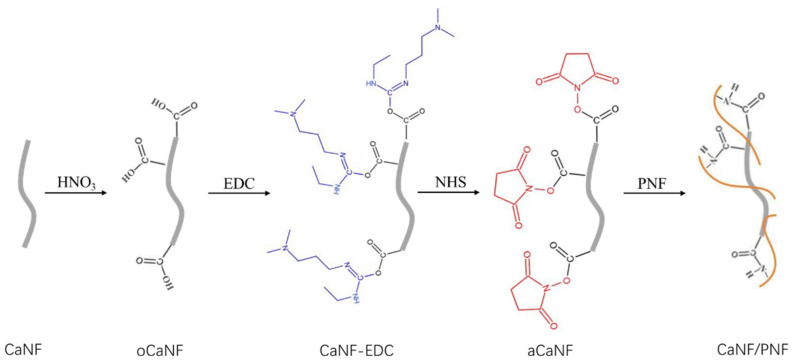
Schematic diagram of the oxidization of CNFs, chemical modification of CaNFs, and chemical binding with PNFs.

The chemical activation process of oCaNFs is shown in Figure 1. In brief, 0.25 g EDC was solved in MES solution to a concentration of 50 mg/mL, and 0.125 g NHS was solved in MES to a concentration of 25 mg/mL After the two solutions were simultaneously added to the above-mentioned 0.1 g oCaNF solid, the mixture was stirred on a magnetic agitator for 24 h. The mixture solution was filtered to get the solid product, which was then washed with water to remove the excess solvent. The final solid product was collected to obtain activated carbon nanofiber black solid. The created activated oxidized carbon nanofibers (aCaNFs) were connected with PNFs via chemical binding.

### 2.3. Tailoring the Formation of Self-Assembled PNFs

The KIIIIKYWYAF peptide used in this work consists of two typical peptide motifs, KIIIIK and YWYAF, in which the KIIIIK sequence can form stable PNFs under suitable reaction conditions. The peptide precursors listed as KIIIIKYWYAF were utilized to form PNFs via molecular self-assembly. In brief, 5 mg peptide powder was dissolved into 1 mL TFE solution, and then 9 mL 0.1% TFA liquid was added to form a uniform peptide solution in a solvent environment with 0.1% TFA/TFE (*v*/*v*, 9:1). The mixed solution was put in a water-bath at 70 °C for inducing the self-assembly of peptide monomers. After a period of time, the peptide solution was taken out for further characterization and synthesis of hybrid materials.

### 2.4. Preparation of CaNF/PNF Nanohybrids and Biomimetic Synthesis of ZrO_2_ NPs

In order to synthesize CaNF/PNF nanohybrids, the activated aCaNF black solid was placed into the above-mentioned 10 mL self-assembled PNF solution with a concentration of 0.5 mg/mL. The mass concentrations of aCaNFs and PNFs in the mixture were set to 10 and 0.5 mg/mL, respectively. The mixed solution was stirred in a magnetic agitator for 24 h. In this way, aCaNFs were fully combined with PNFs to form the CaNF/PNF nanohybrids.

**Figure 2:**
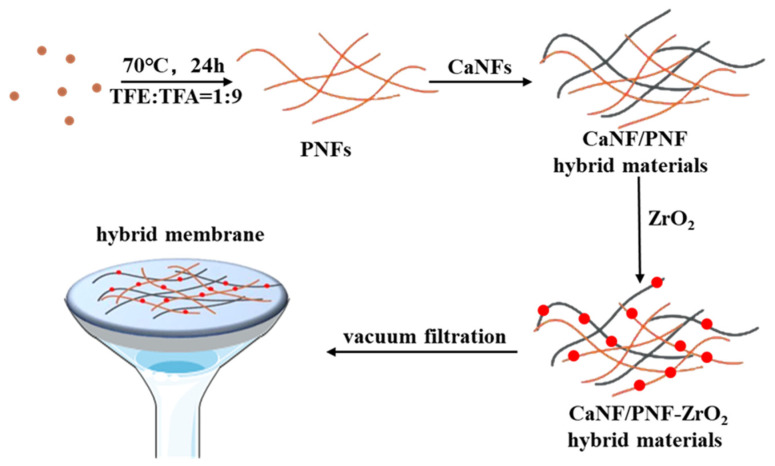
Schematic presentation of biomimetic synthesis of CaNF/PNF-ZrO_2_ hybrid materials and fabrication of hybrid nanofiber membrane by vacuum filtration.

According to the biomineralization method reported by a previous study [5], ZrO_2_ NPs were synthesized by biomimetic mineralization process on the as-prepared CaNF/PNF nanohybrids. In brief, 0.6 g ZrOCl_2_⋅8H_2_O powder was added to the CaNF/PNF nanohybrids under stirring. After 6 h stirring, 0.2 M NaOH aqueous solution was added to the CaNF/PNF nanohybrids drop by drop, meanwhile adjusting the pH value of the solution to 3.5–4 until forming white precipitation. As shown in Figure 2, in order to prepare functional nanohybrid membranes that can be used in the field of defluorination and water purification, the biomimetic mineralized CaNF/PNF-ZrO_2_ nanohybrids were filtered by the vacuum filtration to prepare 2D nanohybrid membranes.

### 2.5. Adsorption Experiments of F^−^ Ions

In the experiment, the operating pressure of rapid filtration was set to about −0.1 MPa, and the aqueous solution containing F^−^ was prepared with 98% NaF solid. For all the adsorption tests, we set the F^−^-containing water samples with a volume of 5 mL, and fix the filtration speed of water samples to approximately 0.17 mL/s. After the filtration, we collected the filtrate solution, which was then measured with the ion chromatograph (IC) to determine the concentration of F^−^ ions accurately.

#### 2.5.1. Effect of the Concentration of PNFs on F^−^ Removal by the Membranes

In order to optimize the conditions for the preparation of nanohybrid membranes, the adsorption efficiency of F^−^ on three kinds of nanohybrid membranes with different PNF content were prepared. The preparation process is as follows:

The activated aCaNF black solid was placed into self-assembled PNF solution (10 mL) with different concentrations (for instance, 0.125, 0.25, and 0.5 mg/mL in this study). The mass concentration of aCaNFs in the mixed solution was set to 10 mg/mL. The mixed solution was stirred on a magnetic agitator for 24 h to fully combine aCaNFs with PNFs to form the CaNF/PNF nanohybrids. Then, under stirring, the solid powder of 0.6 g ZrOCl_2_⋅8H_2_O was added to the CaNF/PNF nanohybrid solution for metallic ion binding. After 6 h stirring, 0.2 M NaOH was used to adjust the solution pH to 3.5–4, until forming white precipitation. After biomimetic mineralization, three kinds of CaNF/PNF-ZrO_2_ nanohybrid membranes with different contents of PNFs, named as the CaNF/PNF-ZrO_2_-0.125, CaNF/PNF-ZrO_2_-0.25, and CaNF/PNF-ZrO_2_-0.5, were fabricated and used to filter the aqueous solution with F^−^ concentration of 50 mg/L quickly, and the removal efficiency of F^−^-containing aqueous solution was compared.

#### 2.5.2. Effect of Different Amounts of ZrO_2_ Precursor on F^−^ Removal by the Membranes

In order to determine the effect of the amount of ZrO_2_ precursor, three kinds of CaNF/PNF-ZrO_2_ nanohybrid membranes were prepared, in which the dosage of ZrOCl_2_⋅8H_2_O was set to 0.3, 0.6, and 1.0 g, respectively. The aqueous solution with F^−^ content of 50 mg/L was filtered rapidly in vacuum, and the best amount of precursor was obtained by comparing the F^−^ removal efficiency of the three membranes.

#### 2.5.3. Adsorption Effect of Membrane on F^−^-Containing Water Samples with Different Concentrations and Adsorption Capacity of the Membranes

The nanofiber hybrid membranes were used as filters to treat different concentrations of F^−^ containing aqueous solution (F^−^ = 50, 100, 200, 300 mg/L) by rapid vacuum filtration.

The CaNF/PNF-ZrO_2_ nanohybrid membranes were placed in 10 mL of F^−^ solution with a concentration of 300 mg/L for 24 h to obtain the adsorption capacity of the membranes towards F^−^.

### 2.6. Sustainability Analysis

The sustainability footprint (SF) analysis of the fabricated membranes was carried out by analyzing 8 evaluation factors, which include the cost, removal efficiency, resources, utilization of natural waste, simplicity, environmental friendliness, degradability, and safety. According to a previous report [33], the overall SF (OSF) value could be calculated through this equation:

$$OSF=100\%\times \overset{8}{\underset{j=1}{\sum }}{\left(\frac{i}{3}\right)}_{j}\times \frac{1}{8}$$

in which “*j*” presents the mentioned 8 sustainability factors, and “*i*” demonstrates the evaluated score of each factor with low (L, *i* = 1), medium (M, *i* = 2), and high (H, *i* = 3). It should be noted that the “Cost” can be indicated as a negative factor for calculating the OSF, which means that the low cost is assigned to a value of *i* = 3, and the high cost is assigned to a smaller value of *i* = 1.

### 2.7. Characterization Techniques

Atomic force microscopy (AFM, FM-Nanoview 6800, Suzhou, China) was utilized to study the self-assembly and formation of PNFs. The AFM tests were conducted with tapping mode in air by using the Tap300Al-G silicon probes. The obtained AFM images were analyzed by the Gwyddion software (Version 2.57). The structure and surface morphologies of CaNFs and hybrid membranes were observed with a transmission electron microscope (TEM, Tecnai G2 F20, FEI Co., Hillsboro, OR, USA) and a scanning electron microscope (SEM, Regulus 8100, Hitachi, Tokyo, Japan). Confocal Raman microscopy was used for the determination of the formation of ZrO_2_ nanoparticles on the membranes after biomimetic synthesis. The element analysis of the synthesized hybrid membranes was conducted on the X-ray photoelectron spectroscopy (XPS, PHI 5000 VersaProbe III, Chigasaki, Japan).

## 3. Results and Discussion

### 3.1. Morphology Characterization of CaNF

**Figure 3:**
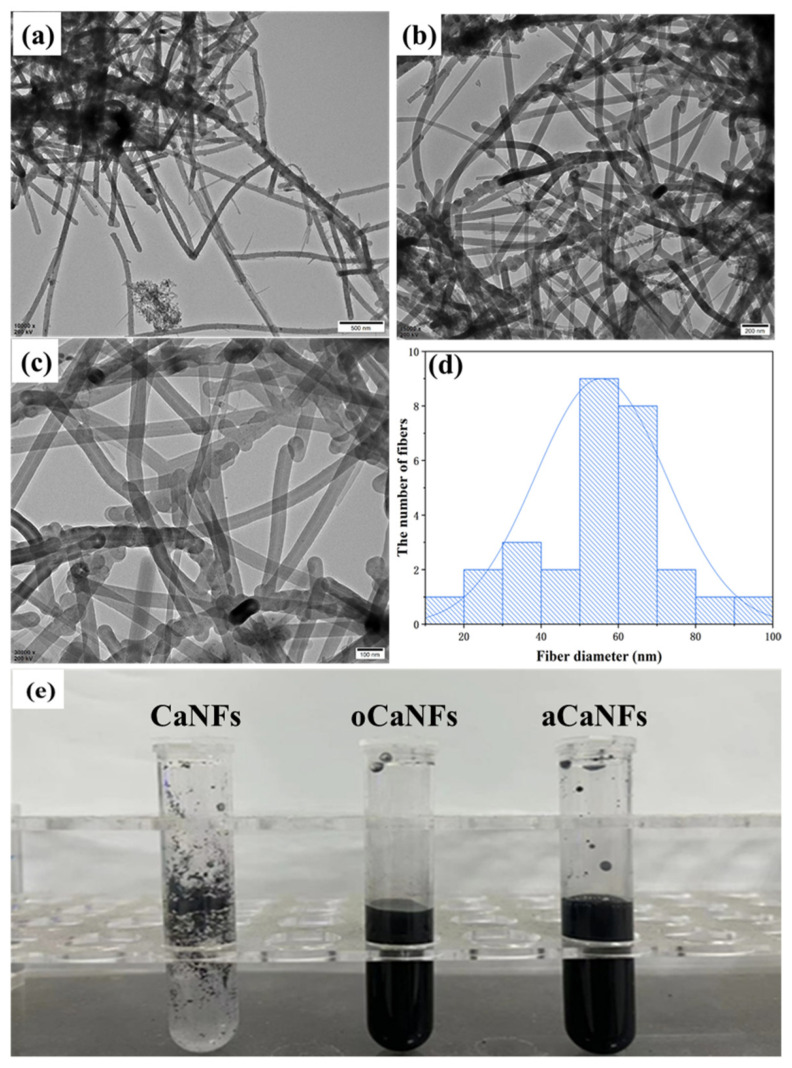
TEM characterizations of (**a**) CaNFs, (**b**) oCaNFs, and (**c**) aCaNFs. The diameter distribution of CaNFs is shown in image (**d**). (**e**) Photographs of chemically modified CaNFs.

Figure 3a shows a typical TEM image of CaNFs, from which it can be seen that the morphology of CaNFs is relatively uniform, and the average diameter of the CaNFs used in this work is located around 60 nm (Figure 3d). During the oxidation of CaNFs to oCaNFs by concentrated HNO_3_, CaNFs were oxidized to form a large number of carboxyl groups. Due to the formation of a large number of carboxyl anions, the solubility of CaNFs in an aqueous solution can be improved, and the electrostatic repulsion between individual CaNFs makes them have a more uniform and dispersed structure. The TEM image of oCaNFs is shown in Figure 3b. After the activation by EDC and NHS, the formed aCaNFs retain the morphology and structure of CaNFs and show similar dispersibility to oCNFs (Figure 3c). Due to the formation of a large number of -COOH groups in the oxidation process, the solubility and dispersion of CaNFs in water improved [19], which can also be proved by simple observation of the solubility and dispersity of CaNFs, oCaNFs, and aCaNFs that shown in Figure 3e. Therefore, the synthesized oCaNFs and aCaNFs exhibit better dispersity in an aqueous solution than pure CaNFs.

### 3.2. Morphology Characterization of PNFs

**Figure 4:**
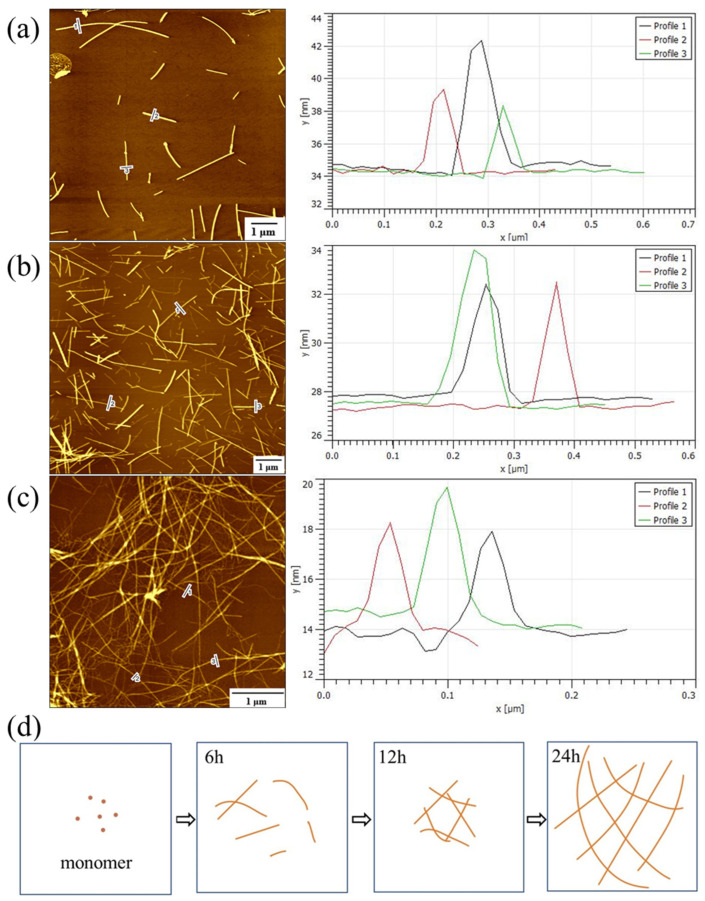
(**a**–**c**) AFM height images and section analysis of PNFs under different reaction times: (**a**) 6 h, (**b**) 12 h, and (**c**) 24 h. (**d**) Formation process of PNFs with different reaction times.

In the beginning, the peptides in the solution are in the monomer state. When the reaction time is 6 h, the number of self-assembled PNFs by peptide monomers is relatively small, and it is obvious that the self-assembly process has not been carried out thoroughly, and the diameter of the fibers is within 10 nm (Figure 4a). When the reaction time is 12 h, the number of PNFs increases, and the diameter of the PNFs is within 10 nm, but the length of the fibers is shorter (Figure 4b). When the reaction time is 24 h, the peptide monomers to form longer PNFs with a uniform diameter within 10 nm (Figure 4c). Based on the above analysis, we suggest that the best reaction time for the formation of the designed PNFs in this study is about 24 h. Based on the obtained AFM data, we proposed a potential formation process of PNFs, as shown in Figure 4d. It is clear that the peptide exhibits a time-dependent growth process from monomers to short protofibrils, nanofibrils, and then long PNFs. In addition, because of the large difference in the diameter range of the formed PNFs (10 nm) and the used CaNFs (60 nm), the PNFs can be well distinguished from the formed CaNF/PNF nanohybrids in this study.

### 3.3. Characterization of CaNF/PNF Hybrid Materials

After two functional nano-building blocks, PNFs and CaNFs, were prepared, the CaNF/PNF nano-hybrid materials composed of PNFs and CaNFs could be further prepared by mixing them directly. Previous studies have shown that motif-designed PNFs can be used as nanoscale components to synthesize functional hybrid nanomaterials [5,38]. In this work, self-assembled PNFs and CaNFs are combined together to form a kind of nanofiber hybrid material through both electrostatic interaction and chemical binding, in which the formed CaNF/PNF hybrids provide binding sites and nanoscale support for the mineralized ZrO_2_ NPs.

**Figure 5:**
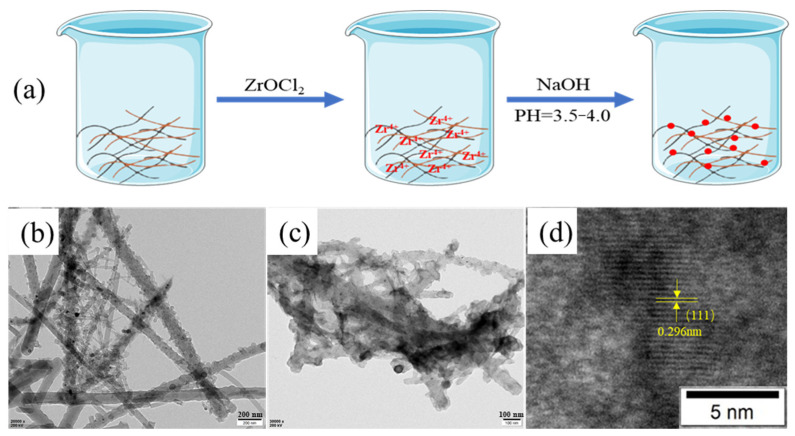
Biomimetic synthesis of ZrO_2_ nanoparticles on CaNF/PNF hybrids: (**a**) schematic presentation of the green synthesis process. (**b**,**c**) Typical TEM images of CaNF/PNF nanohybrids before and after the formation of ZrO_2_. (**d**) HR-TEM image of ZrO_2_ NPs on CaNF/PNF-ZrO_2_ minerals.

In the biomimetic synthesis process, positively charged Zr^4+^ binds with negatively charged groups and amino groups on CaNFs and PNFs through non-covalent interaction, which mediates the formation of ZrO_2_ NPs by adjusting the pH value of the solution between 3.5 and 4 (Figure 5a). The CaNF/PNF nano-hybrid materials before and after biomimetic mineralization were characterized by TEM. From the TEM image (Figure 5b) of the CaNF/PNF nano-hybrid materials before mineralization, it can be seen that a large number of PNFs and CaNFs are combined. After the biomimetic mineralization, a large number of ZrO_2_ NPs (Figure 5c) are formed on the CaNF/PNF nanohybrids. The corresponding HR-TEM image (Figure 5d) shows that the lattice spacing of the generated nanoparticles was about 0.296 nm, which is consistent with previously reported crystal plane data of the synthesizedZrO_2_ NPs [5]. According to the obtained results, it can be found that both PNFs and CaNFs play important role in guiding the crystal nucleation and final formation of ZrO_2_ NPs during the biomimetic mineralization of the hybrid materials. Compared with other methods for the preparation of ZrO_2_ NPs, such as hydrothermal synthesis [39], sol-gel synthesis [40], and microwave-assisted combustion [41], the biomimetic mineralization method in this work has the advantages of simplicity, economy, and green, and shows more application prospects in many fields.

### 3.4. Characterization of Organic-Inorganic Hybrid Membranes

**Figure 6:**
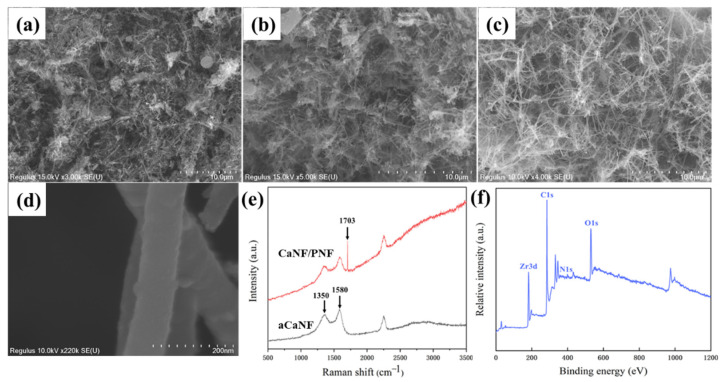
(**a**–**c**) SEM images of the organic-inorganic hybrid membranes with PNFs amount of (**a**) 0.125, (**b**) 0.25, and (**c**) 0.5 mg. (**d**) Enlarged SEM image of the membrane, (**e**) Raman spectra of aCaNF and CaNF/PNF membranes, and (**f**) XPS spectrum of the biomimetic CaNF/PNF-ZrO_2_ membrane.

The amount of CaNFs and PNFs are important for the fabrication of organic-inorganic hybrid membranes. Figure 6a–c show the cross-sectional SEM images of the hybrid membranes that were prepared with 0.1 g CaNFs and adjusted PNF dosage of 0.125, 0.25, and 0.5 g, respectively. It can be seen from the SEM images that when the peptide dosage was 0.5 mg, the prepared hybrid membrane has a compact porous network structure, which can make the membrane have a larger surface area and more binding sites, further promoting the adsorption of F^−^ with enhanced performance. So chose the amount of PNFs as 0.5 mg to prepare the hybrid membrane for the next water purification. From the enlarged SEM image (Figure 6d), it can be observed that ZrO_2_ NPs are formed along the single fiber, and the size of the biomimetic nanoparticles was about 20 nm.

The aCaNF and CaNF/PNF membranes were further characterized by Raman and XPS spectroscopy. As shown in Figure 6e, two strong peaks at 1350 and 1580 cm^−1^ are attributed to CaNF graphitization-induced G-band and disorder-induced D-band [21], indicating the existence of aCaNFs clearly. After the combination of CaNFs and PNFs, the prepared CaNF/PNF hybrid membrane reveals a new strong peak at 1703 cm^−1^, which is ascribed to the amide I of PNFs [42]. Therefore, we suggest the obtained Raman spectroscopy data proves that the CaNF/PNF hybrid membrane has been prepared successfully. In addition, from the XPS spectrum, it can be found that the biomimetic CaNF/PNF-ZrO_2_ hybrid membrane has C1s, N1s, O1s, and Zr3d peaks (Figure 6f). Based on the above characterizations of various membranes, we suggest that the CaNF/PNF-ZrO_2_ organic-inorganic hybrid membranes were fabricated successfully.

### 3.5. F^−^ Ion Adsorption of Organic-Inorganic Hybrid Membranes

**Figure 7:**
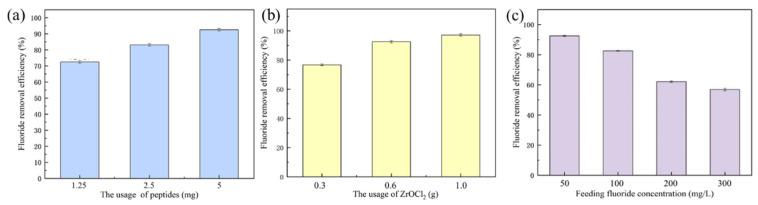
F^−^ ion adsorption experiments: (**a**) removal efficiency of biomimetic inorganic-organic hybrid membranes with various PNF dosages, (**b**) removal efficiency of biomimetic inorganic-organic hybrid membranes with various ZrO_2_ loading, and (**c**) Removal efficiency of membranes towards F^−^-containing samples.

After the preparation and characterizations of the CaNF/PNF/-ZrO_2_ hybrid membranes, we further studied the F^−^ removal performance of the hybrid membranes. First of all, the effects of different amounts of PNFs (1.25, 2.5, and 5 mg) on the F^−^ removal performance of the membranes were studied. The adsorption data in Figure 7a indicates that the F^−^ removal efficiency (92.54%) when the dosage of PNF was 5 mg was significantly higher than that of 1.25 (72.47%) and 2.5 mg (83.07%). Then, the effect of the amount of ZrO_2_ precursor on the defluorination efficiency of the membranes was also studied. As shown in Figure 7b, when the F^−^ solution of 50 mg/L was filtered rapidly, the hybrid membrane with the dosage of 0.3, 0.6, and 1.0 g ZrO_2_ precursors could obtain 76.74%, 92.54%, and 97.19% removal efficiency, respectively. Considering the cost and energy consumption, the best dosage of ZrO_2_ precursor was selected as 0.6 g.

Finally, the removing performance of the fabricated membranes towards F^−^ was tested after rapid filtration in water samples with different F^−^ concentrations (from 20 to 300 mg/L). It can be found that when the F^−^ concentration is lower than 100 mg/L, an efficiency of more than 80% is achieved (Figure 7c). When the concentration of F^−^ is increased to 200 mg/L, the treatment efficiency of the fabricated inorganic-organic hybrid membranes can be maintained at more than 62%. However, in the case of high F^−^ concentration (300 mg/L), the removal efficiency decreased significantly to about 57%. In addition, by fully adsorbing the hybrid membrane in F^−^-containing an aqueous solution, the adsorption capacity of the hybrid membrane is 10.40 mg/g. Based on the adsorption experiment, we suggest that the biomimetic CaNF/PNF/-ZrO_2_ hybrid membranes exhibit a good efficiency in rapid filtration and purification of water samples containing F^−^.

### 3.6. Sustainability Analysis

Sustainability is an important factor to evaluate the properties of materials [43], especially on the point of materials for environmental science applications. In order to evaluate the sustainability of the fabricated inorganic-organic hybrid membranes in this study, we utilized the OSF method to analyze the performance of the formed membranes. Through comparing with the other two removal methods of F^−^, such as the electrodialysis method [44] and chemical deposition [45], the SF comparison and the OSF values of three removal methods for F^−^ were obtained.

**Figure 8:**
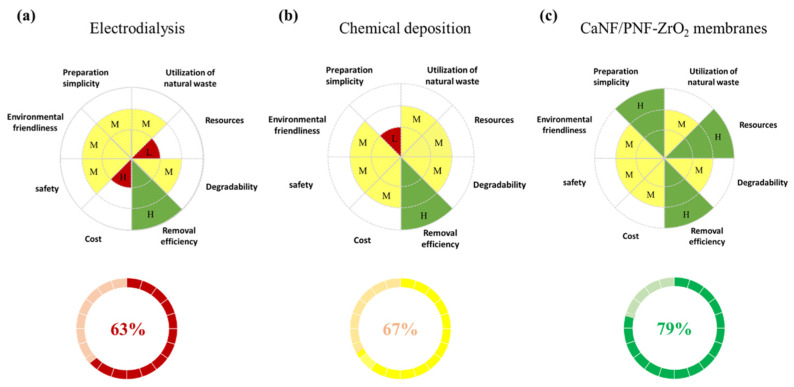
Sustainability analysis of inorganic-organic hybrid membranes by comparing with two adsorption methods: (**a**) Electrodialysis method [44], (**b**) Chemical deposition method [45], and (**c**) hybrid membrane filtration.

Figure 8 shows the SF analysis of the created inorganic-organic hybrid membranes by comparing with two other F^−^ removal methods. Through analyzing eight sustainability factors of three methods, we found that the hybrid membrane prepared in this work has superiority in some sustainability factors compared to the other two methods. The OSF values of the electrodialysis method, chemical deposition method, and adsorption method based on CaNF/PNF-ZrO_2_ membranes are calculated to be 63%, 67%, and 79%, respectively. Based on this SF analysis, we suggest that the CaNF/PNF-ZrO_2_ membranes exhibit better sustainability than the other two methods by considering these sustainability factors. The fabrication of membranes needed lower costs and energy consumption. In addition, the membranes in this study revealed rapid adsorption of F^−^, which provides a feasible way for practical water purification application.

## 4. Conclusions

In summary, we report the preparation of hybrid nanomaterials by chemical binding of CaNFs and PNFs, and the biomimetic mineralization of ZrO_2_ NPs on nanofibers to prepare functional CaNF/PNF-ZrO_2_ nano-hybrid materials. The fabricated CaNF/PNF-ZrO_2_ nano-hybrid membranes were prepared successfully by the vacuum filtration method. In addition, by comparing the defluorination effect, the optimal dosages of PNFs and ZrO_2_ precursor were determined to be 5 mg and 0.6 g, respectively. The results of adsorption tests indicated that the hybrid nanofiber membranes have good adsorption and removal performances for F^−^ from water samples. The treatment efficiency of the created inorganic-organic hybrid membranes kept more than 80% under relatively low F^−^ concentrations (<100 mg/L), and the saturated adsorption test indicated that the fabricated hybrid membrane has a high adsorption capacity (10.40 mg/g). It was found that the use of CaNFs greatly reduced the cost of the membranes, which provides a new idea for sustainable, economic, rapid, and efficient F^−^ removal. Furthermore, we suggest that it is possible to increase the adsorption capacity of membranes by forming more ZrO_2_ NPs on the hybrid membrane via biomineralization. In addition, the sustainability analysis of the designed membranes was carried out using the SF method by comparing with the other two removal methods of F^−^. the hybrid membranes of this work had an OSF value of 79%, which is higher than the electrodialysis method (63%) and chemical deposition method (67%). Therefore, the hybrid membranes have a good sustainable performance, and exhibit high potential for the removing of F^−^ from sewage.

## Data Availability

Not applicable.
